# Preparation, Thermal, and Mechanical Characterization of UV-Cured Polymer Biocomposites with Lignin

**DOI:** 10.3390/polym12051159

**Published:** 2020-05-19

**Authors:** Marta Goliszek, Beata Podkościelna, Tomasz Klepka, Olena Sevastyanova

**Affiliations:** 1 Department of Polymer Chemistry, Institute of Chemical Science, Faculty of Chemistry, Maria Curie-Sklodowska University, M. Curie-Sklodowska Sq. 5, 20-031 Lublin, Poland; beatapod@poczta.umcs.lublin.pl; 2 Analytical Laboratory, Institute of Chemical Science, Faculty of Chemistry, Maria Curie-Sklodowska University, M. Curie-Sklodowska Sq. 5, 20-031 Lublin, Poland; 3 Department of Technology and Polymer Processing, Faculty of Mechanical Engineering, Lublin University of Technology, Nadbystrzycka 36, 20-618 Lublin, Poland; t.klepka@pollub.pl; 4 Department of Fibre and Polymer Technology, KTH Royal Institute of Technology, Teknikringen 56-58, SE-10044 Stockholm, Sweden; olena@kth.se; 5 Wallenberg Wood Science Center (WWSC), Department of Fibre and Polymer Technology, KTH Royal Institute of Technology, Teknikringen 56-58, SE-10044 Stockholm, Sweden

**Keywords:** lignin, UV-cured, biocomposites, thermal properties, TG/FTIR, DSC

## Abstract

The preparation and the thermal and mechanical characteristics of lignin-containing polymer biocomposites were studied. Bisphenol A glycerolate (1 glycerol/phenol) diacrylate (BPA.GDA) was used as the main monomer, and butyl acrylate (BA), 2-ethylhexyl acrylate (EHA) or styrene (St) was used as the reactive diluent. Unmodified lignin (L) or lignin modified with methacryloyl chloride (L-M) was applied as an ecofriendly component. The influences of the lignin, its modification, and of the type of reactive diluent on the properties of the composites were investigated. In the biocomposites with unmodified lignin, the lignin mainly acted as a filler, and it seemed that interactions occurred between the hydroxyl groups of the lignin and the carbonyl groups of the acrylates. When methacrylated lignin was applied, it seemed to take part in the creation of a polymer network. When styrene was added as a reactive diluent, the biocomposites had a more homogeneous structure, and their thermal resistance was higher than those with acrylate monomers. The use of lignin and its methacrylic derivative as a component in polymer composites promotes sustainability in the plastics industry and can have a positive influence on environmental problems related to waste generation.

## 1. Introduction

Epoxy resins are among the most important materials in the modern polymer industry [1,2,3,4,5,6,7,8,9,10]. They can be further reacted to form thermoset polymers with a strong adherence to many substrates, a great thermal resistance, good electrical and mechanical properties, a low order of shrinkage on cure, a high degree of chemical and solvent resistance, and a moderate cost [11,12,13,14]. They are used for protective coatings and finishes, adhesives, automobile parts, metal jar lids, food-contact surface lacquer coatings for cans, as a coating for PVC pipes and in aerospace applications [15]. To synthesize the thermoset through a free-radical mechanism, the epoxy resins must contain unsaturated groups. In some cases, epoxy resins can be esterified with acrylic acid to produce diacrylate esters, also called vinyl ester resins (e.g., bisphenol A glycerolate (1 glycerol/phenol) diacrylate). Difunctional acrylates are used as UV-curable cross-linking components of varnishes, inks, coatings, and lacquers. They are characterized by a high viscosity, and they must be diluted with a low molecular weight co-monomer (e.g., styrene and acrylate monomers). Curing occurs through free radical chain-growth copolymerization between the unsaturated vinylene molecules and the co-monomer, where the co-monomer acts as an agent to link the adjacent vinylester chains [16,17,18]. 

To promote the sustainability of thermoset polymers and of the plastic industry in general, renewable materials may be incorporated as fillers or substitutes for synthetic ones [19]. Various natural polymers have been used or tested as fillers in composite materials in recent years [20,21,22,23,24]. The unquestionable advantages of natural organic fillers are their renewability, availability, and relatively low cost, all of which contribute to a low price for the final product [25]. The use of renewable eco-fillers in composite materials, especially from the agricultural or forest industries, is therefore of great interest.

One of the most promising natural materials with a great potential for improving the properties of polymer composites is lignin [26]. It is one of the main components of wood and the second most abundant polymer in nature. Its aromatic nature and functionality make it a desirable component in many polymeric formulations. Industrial lignins are by-products of pulping processes and bio-ethanol production [27], and this makes them a cheap and widely available biopolymer.

The chemical structure of native lignin has been thoroughly investigated by spectroscopic and chemical methods [28,29,30,31]. It is a complex polyphenolic material containing various polar (alcoholic and phenolic hydroxyl, ether, and methoxyl) functionalities. It has an amorphous structure, which arises from the enzyme-initiated dehydrogenative polymerization of the phenylpropane units coniferyl, p-coumaryl, and sinapyl alcohols [32,33]. The exact chemical structure of lignin remains undefined, because its chemical composition depends on the source and on the employed isolation method [30,34,35,36,37]. 

One of the possible applications of lignin is its incorporation into polymeric materials. Since each monomeric lignin unit usually has more than two hydroxyl groups, lignin-based materials such as polycaprolactone derivatives, polyurethane derivatives, and epoxy resins can be obtained by using the hydroxyl group as the reaction site [38,39]. Lignin can be applied as a reinforcing filler, although its naturally variable composition and irregular structure can lead to a reduced toughness, while its phenolic moieties can introduce heat instability [26,40]. It nevertheless has benefits, such as cost reduction, sustainability, and improved stiffness. Košíková et al. [41] examined lignin’s stabilizing effect on the thermal degradation of a natural rubber that had sulfur-free lignin as a natural filler. They showed that, when used as a filler, lignin increased the resistance of natural rubber vulcanizates to thermo-oxidative degradation in air. Strzemiecka et al. [42] prepared and characterized lignin–SiO_2_ hybrid fillers as potential binders for phenolic resins. Sun et al. [43] prepared novel lignin epoxy composites via the covalent incorporation of depolymerized lignin epoxide as a submicron filler in an epoxy matrix and showed that the composites had better mechanical properties than neat epoxy. Goliszek et al. [44] investigated the accelerated aging of lignin-containing polymer composites of styrene and methyl methacrylate. Wood et al. [45] used lignin as a compatibilizer in hemp–epoxy composite samples and showed that the addition of lignin improved the structural properties of the obtained composites.

The thermal behavior of lignin is important for making processable lignin-containing materials with good final properties. According to Sen et al. [46], lignin can act as a thermosetting and a thermoplastic material. It is thermoplastic because of its chemical structure and inter- and intra-molecular hydrogen bonds. Lignin can also act as a thermosetting material because it forms cross-linked structures at elevated temperatures, and its molecular weight dramatically increases due to radical-initiated self-polymerization. Hajirahimkhan et al. [47] prepared UV-cured coatings with up to 31 wt % of methacrylated lignin in formulations. These coatings showed a high thermal stability/char formation and a high hydrophobicity.

In the present work, biocomposites with lignin and a vinylester resin based on bisphenol A were prepared and characterized. This epoxy resin was chosen as the matrix because it is used in various industrial applications and therefore possesses industrially relevant properties. Lignin was selected as the reinforcement because of its cost effectiveness, natural origin, and availability. Lignin, unmodified or esterified with methacryloyl chloride, was polymerized with a bifunctional matrix to create a polymer network. The thermal properties of the biocomposites, as well as their volatile decomposition products, were investigated in detail. 

## 2. Materials and Methods

### 2.1. Chemicals

Bisphenol A glycerolate (1 glycerol/phenol) diacrylate, butyl acrylate, 2-ethylhexyl acrylate, styrene, kraft lignin, α,α’-Azoiso-bis-butyronitrile (AIBN), and 2,2-dimethoxy-2-phenylacetophenone were obtained from Sigma-Aldrich. Modified lignin was obtained according to the procedure described in [48]. Briefly, the lignin sample and methylene chloride were placed in an ice bath with triethylamine and stirred. Methacryloyl chloride was then added dropwise, and the reaction was allowed to proceed for 1 h at 5 °C and for an additional hour at room temperature. The obtained material was filtered off, washed three times with water to remove trimethylamine hydrochloride, and extracted with methylene chloride.

### 2.2. Preparation of Biocomposites 

The appropriate amount of bisphenol A glycerolate (1 glycerol/phenol) diacrylate (BPA.GDA) was weighed into a glass vessel. Butyl acrylate (BA), 2-ethylhexyl acrylate (EHA), or styrene (St) was then added dropwise while stirring. The ratio of BPA.GDA to BA/EHA/St was 10:3. An adequate amount of unmodified (L) or methacrylated (L-M) lignin (5 or 10 wt %) was then added, and stirring was continued to obtain a homogeneously dispersed mixture. Finally, 4 wt % of 2,2-dimethoxy-2-phenylacetophenone (IQ) as a photoinitiator was added, and, after stirring, the mixture was placed in a glass vessel and cured under a UV lamp for 0.5 h [49]. For comparison, composites without lignin were also synthesized. For the dynamic mechanical analyzer (DMA) and mechanical tests, when a thick sample (more than 3 mm) was needed, the preparation was modified and an additional thermal initiator (AIBN 1 wt %) was used. After the initial irradiation process (30 min), the composites were transferred to an oven and heated for 3 h at 80 °C for additional crosslinking reactions. The parameters of the syntheses are listed in Table 1.

### 2.3. Characterization Methods

The attenuated total reflection (ATR) was recorded using infrared Fourier transform spectroscopy on a TENSOR 27 Bruker spectrometer equipped with a diamond crystal (Ettlingen, Germany). The spectra were recorded in the range of 600–4000 cm^−1^ with 32 scans per spectrum at a resolution of 4 cm^−1^.

The calorimetric measurements were carried out with a DSC 204 Netzsch calorimeter (Selb, Germany) operated in a dynamic mode. The dynamic scans were performed at a heating rate of 10 °C·min^−1^, the first scan being from 20 °C to a maximum of 110 °C to remove adsorbed moisture and the second being from 25 to 550 °C in a nitrogen atmosphere (30 cm^3^·min^−1^). The mass of the sample was between 5 and 10 mg. An empty aluminum crucible was used as reference. 

Thermal analysis was conducted using an STA 449 Jupiter F1, Netzsch (Selb, Germany). The samples were heated from 30 to 800 °C at a rate of 10 °C·min^−1^ in a dynamic atmosphere of helium (25 cm^3^·min^−1^). An S TG–DSC type sensor thermocouple of was used with an empty Al_2_O_3_ crucible as a reference. The volatile decomposition products were detected and identified using a TGA 585 FTIR spectrometer (Bruker, Germany), the FTIR spectra being recorded from 600 to 4000 min^−1^ with a resolution of 4 min^−1^.

The hardness of the materials was measured by the Shore D method using a 7206/H04 analog hardness testing apparatus, Zwick (Ulm, Germany) at 23 °C. Readings were taken after 15 s. 

Thermomechanical properties were determined using the DMA Q800 from TA Instruments equipped with a double-cantilever device. Samples with dimensions of 60 mm × 10 mm × 2 mm were tested. The temperature scanning was carried out from 0 to 200 °C with a constant heating rate of 3 °C·min^−1^ at a sinusoidal distortion of 10 µm amplitude and a frequency of 1 Hz frequency. The glass-transition temperature, damping factor, storage modulus, and loss modulus were determined.

Specimens of the materials were subjected to uniaxial tensile strength (ISO 527, ASTM D-1798 standard) and three-point bending tests (ISO 170, ASTM D-790 standards). The tests were carried out using a Zwick/Roell Z010 universal tensile-testing machine (Ulm, Germany) with a test speed of 50 mm/min at 23 °C. Specimens were cut from a pressed sheet that was 2 mm thick, 10 mm wide, and 60 mm long.

## 3. Results and Discussion

**Figure 1:**
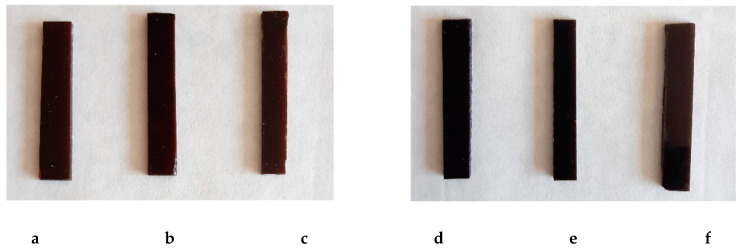
Photographs of the composites: (**a**) EHA + 10% L-M; (**b**) BA + 10% L-M; (**c**) St + 10% L-M; (**d**) EHA + 10% L; (**e**) BA + 10% L; and (**f**) St + 10% L.

When a mixture of BPA.GDA, reactive diluent (either BA, or EHA, or St), and unmodified lignin was subjected to UV radiation in the presence of photoinitiator, composites of various thickness could easily be prepared despite the well-known radical scavenging and UV-inhibiting properties of the phenolic groups in lignin [50,51,52,53,54,55,56] (Figure 1d–f). 

When methacrylated lignin, in which most of the phenolic groups are substituted by vinyl groups, was used as a component of the system, UV-initiated polymerization readily occurred on the surface of the composite, leaving the core unreacted. This phenomenon was observed for a wide quantity range of the photoinitiator. It seems that the polymerized surface layer prevented the penetration of UV radiation into the bulk of the composite and thus inhibited complete curing. Only thin films could thus be produced by the photopolymerization of systems containing methacrylated lignin. To be able to produce the thicker samples needed for the mechanical testing, an additional thermal cross-linking step in the presence of AIBN (1 wt %) was performed. The obtained samples are shown in Figure 1a–c.

### 3.1. Structural Characterization

The ATR/FT-IR spectra of the composites are shown in Figure S1a–c (Supplementary Material). The broad absorption band with a maximum at 3453 cm^−1^ in all the spectra indicated the presence of –OH groups in the organic filler [20] and of hydroxyl groups in the structure of BPA.GDA [57]. The intensity of this band increased with increasing content of lignin in the biocomposite and was highest for the materials with 10 wt % of unmodified lignin (L). As expected, it was lower for the biocomposites with modified lignin, where hydroxyl groups had reacted with methacryloyl chloride [48]. The observed peaks at 2961 and 2873 cm^−1^ were due to C–H stretching in the methyl and methylene groups [58]. Their intensities usually increased with the increasing content of lignin, indicating that these groups, present in the lignin structure, were incorporated into the biocomposites. These signals were also more intense for the composites with EHA than for those containing BA, and they were less intense for the composites with St. The signal at 1726 cm^−1^ corresponded to C=O stretching in ester groups [57]. The intensity of this signal also increased with increasing lignin content, and it was more intense in the biocomposites with methacrylated lignin (L-M) [48] and in the materials where acrylate monomers were used as reactive diluents (Figure S1a–b, Supplementary Materials) [59]. The peaks at 1607 and 1509 cm^−1^ were due to the stretching vibrations of –C=C in benzene rings and aromatic skeletal vibrations [20]. They increased in intensity, showing that the aromatic systems of lignin were incorporated into the biocomposites. The signal at 1461 cm^−1^ corresponded to the C–H asymmetrical deformations in the –CH_3_ and –CH_2_– groups. The bands at 1235 and 1181 cm^−1^ were due to C–O stretching vibrations in acrylates [60] and were more intense for the biocomposites with L-M. The signal at 1040 cm^−1^ was due to the C–O–C stretching in ethers, whereas the peak at 829 cm^−1^ was characteristic of 1,4-substituted aromatic rings [61]. Among the biocomposites with styrene (Figure S1c, Supplementary Materials), a band at 701 cm^−1^ due to the bending of the aromatic carbon-hydrogen bonds of styrene was also observed [62].

### 3.2. Thermal Stability

**Figure 2:**
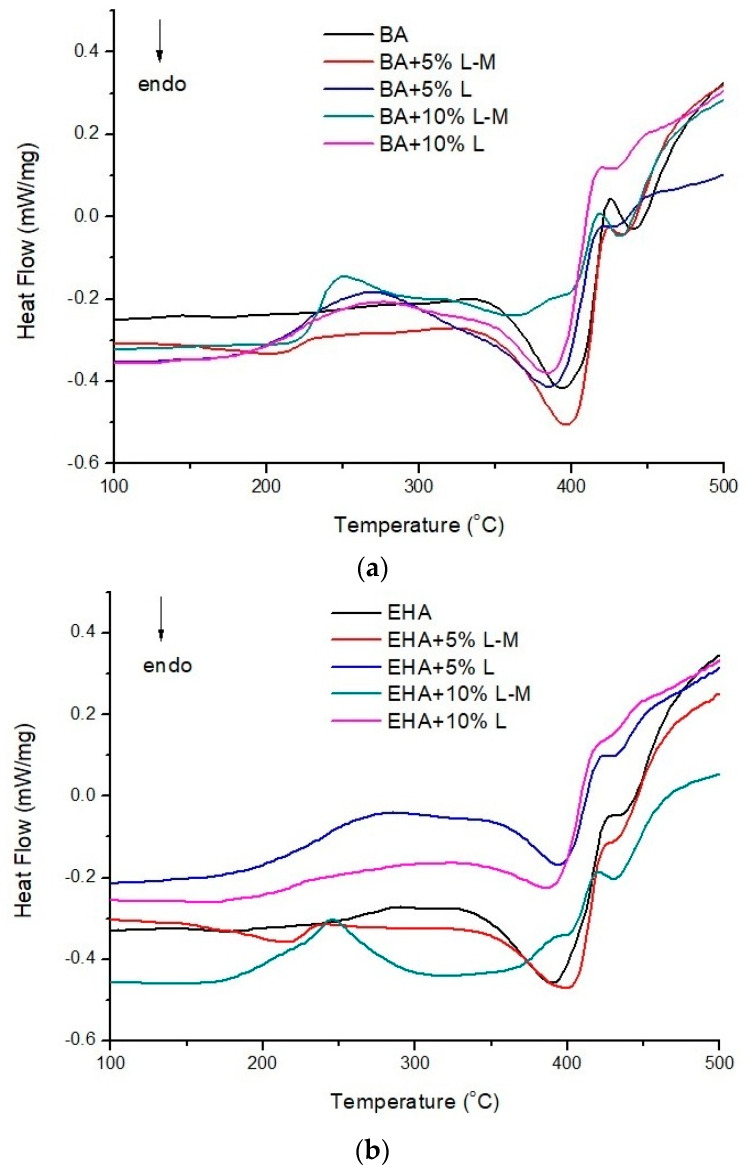

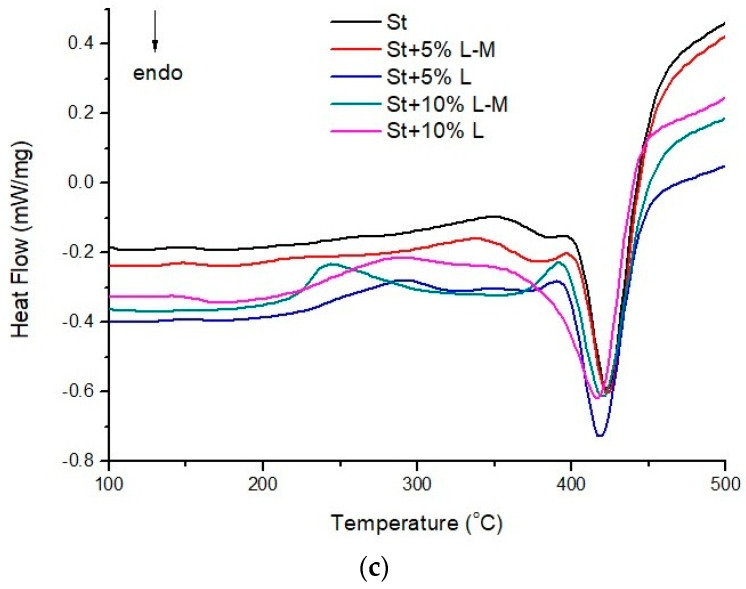
DSC curves of: (**a**) biocomposites with BA, (**b**) biocomposites with EHA, and (**c**) biocomposites with St.

To study the thermal behavior of lignin-containing materials, DSC analysis was carried out. The DSC curves for the biocomposites are shown in Figure 2a–c. Two endothermic peaks were evident in the 350–460 °C range for the composites with acrylate monomers (Figure 2a–b), and one endothermic peak in the 390–450 °C range was evident for the composites with styrene (Figure 2c). These peaks are associated with the total thermal degradation of materials. Among the composites with acrylate monomers, which are less reactive than those with a styrene monomer, homopolymerization and cyclization processes can take place [44] and can thus result in an increase in material heterogeneity. An exothermic signal was visible at 250 °C for all the materials containing 10% modified lignin (BA + 10% L-M and EHA + 10% L-M), and it could be assigned to the further polymerization processes of unreacted L-M. It was most visible in the biocomposites with acrylates, and the peak was much smaller in St + 10% L-M. Among biocomposites with 5% L-M, no such effect was observed. When 5% of modified lignin was used, it could take part in the creation of a polymer network and thus prevent the homopolymerization of the monomers. The addition of 5% L-M led to a slight increase in the total thermal degradation temperature, when the L-M was increased to 10%, the polymerization of the unreacted L-M occurred. In biocomposites with L, interactions may have occurred between the hydroxyl groups of lignin and the carbonyl groups of acrylates. The curves for styrene-containing materials were more sharp than those for the materials containing acrylate. These observations also suggested that the styrene monomer was the most reactive, resulting in more crosslinked and homogeneous materials.

Calorimetric measurements were also carried out in the temperature range from −20 to 200 °C, before and after the polymerization process, for the samples with the highest proportion of lignin (10 wt %). The curves are presented in Figure S2a–c. For the mixture of monomers (BA/EHA/St + 10% L-M_liq._), a single well-shaped exothermic effect in the temperature range of 70–130 °C was clearly visible (Figure S2a, Supplementary Materials). It was related to the process of polymerization. Among the tested polymers (Figure S2b–c, Supplementary Materials), only small exothermic effects were observed at ca 70 °C. For the polymers containing L-M (Figure S2c, Supplementary Materials), wide exothermic effects were also observed around 250 °C; these were related to further post-polymerization reactions, what could indicate that the polymerization process was hindered by L-M. 

The thermal behavior of the biocomposites with the largest content of lignin (10%) and of the corresponding composites without lignin was studied by thermogravimetric analysis, as shown in Figures S3–S5 (Supplementary Materials). Table 2 presents the temperatures of 5% and 50% mass loss (*T*_5%_ and *T*_50%_, respectively), as well as the temperature of maximum mass loss (*T*_max_) with the mass losses (*m*_loss_) in each decomposition stage and the residual masses (RM). Up to a temperature of ca. 300 °C, all the composites without lignin were thermally stable in an inert atmosphere of helium. Further heating led to their decomposition in a single step. A separate peak was observed in the DTG curves, showing the total thermal degradation of the polymer networks. 

Among the lignin-containing materials, the decomposition of BA + 10% L-M took place in two stages. The first 8.01% mass loss (*m*_loss1_) at 182 °C (*T*_max1_) was assigned to the thermal decomposition of unreacted monomers and unreacted methacrylated lignin. The second 83.14% mass loss (*m*_loss2_) at 418 °C (*T*_max2_) was probably related to the total decomposition of the polymer network. The thermal decomposition of BA + 10% L took place in a single step, but *T*_max2_ was lower (410 °C), probably because lignin acted as a filler and was a part of the polymer network. 

A similar situation was observed for the biocomposites with EHA. The decomposition of EHA + 10% L took place in two stages—the first stage at *T*_max1_ 186 °C and *m*_loss1_ 8.64% probably being related to the decomposition of small lignin molecules. In the case of EHA + 10% L-M, the first decomposition stage was at *T*_max1_ 191 °C and *m*_loss1_ 11.47%. The *T*_max2_ temperatures were 417 and 411 °C for EHA + 10% L-M and EHA + 10% L, respectively, which were slightly lower than the *T*_max2_ of the composite without lignin (425 °C). 

St + 10% L degraded in a single step, and the small effect at 78 °C was assigned to the evaporation of residual moisture and not to the degradation of the composite. The thermal decomposition of St + 10% L-M took place in two steps, but the first step at *T*_max1_ 164 °C was related to *m*_loss1_ 5.87%, which was much lower than the m_loss1_ value for the corresponding biocomposite with acrylate monomers. When styrene was used as a reactive diluent, more modified lignin could be built into the polymer network. The *T*_max2_ values for the St + 10% L-M and for the composite without lignin were very similar (423 and 422 °C, respectively), and the *T*_max2_ for the St + 10% L was only slightly lower (419 °C). The higher thermal resistance of biocomposites with St could be assigned to the aromatic chemical structure of this monomer.

The final residue (RM) evaluated at 800 °C for the reference composites without lignin was the highest for BA and the lowest for St. After the addition of lignin, the highest increase in the RM value was observed in the St-system. The differences in RM values between systems with L and L-M were due to differences in the chemical structures of the used lignin samples. 

**Figure 3:**
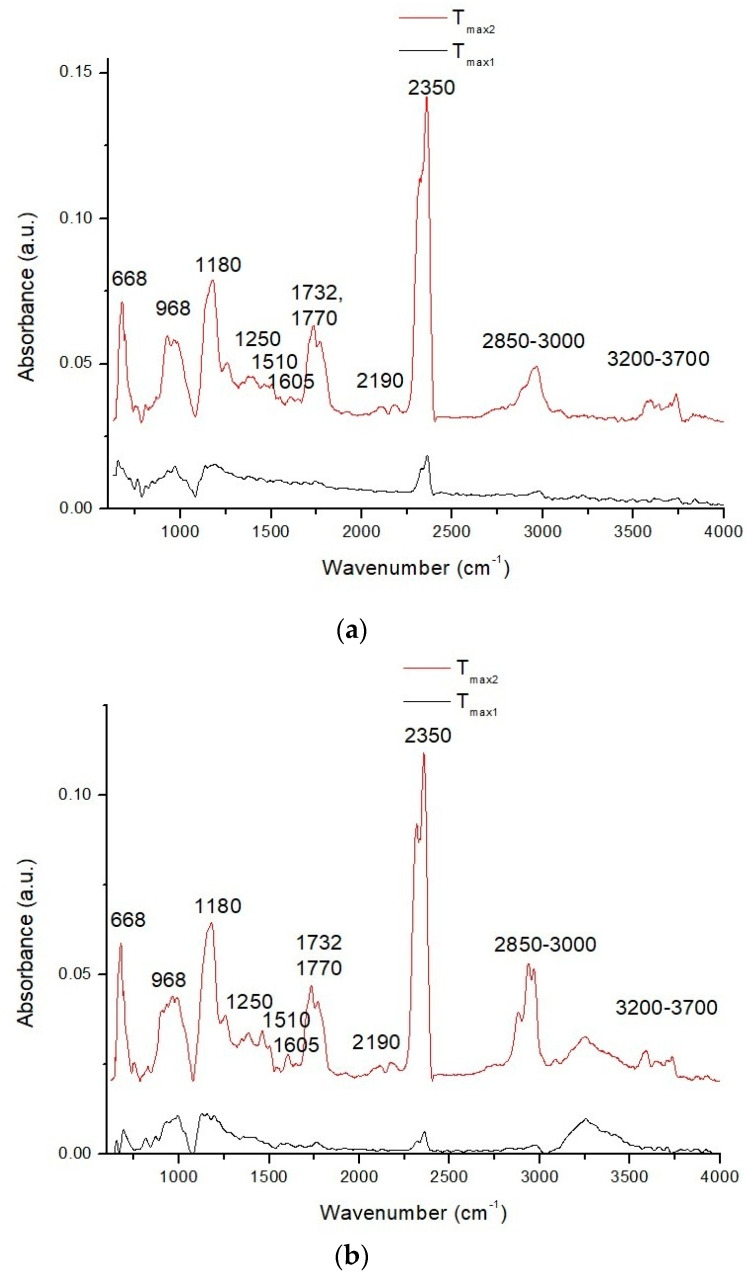

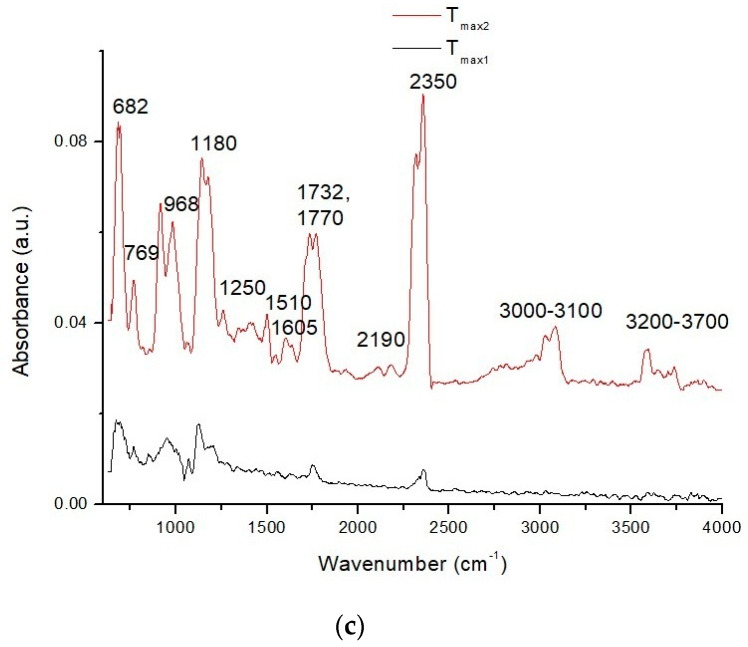
FTIR spectra of the volatile decomposition products of (**a**) BA + 10% L-M, (**b**) EHA + 10% L-M, and (**c**) St + 10% L-M.

In order to conduct a deeper analysis of the thermal degradation of the biocomposites containing methacrylated lignin, the FTIR spectra of the gases evolved from BA + 10% L-M, EHA + 10% L-M, and St + 10% L-M during *T*_max1_ and *T*_max2_ were detected and are shown in Figure 3a–c. 

In the first decomposition stage of BA + 10% L-M (Figure 3a), bands at 2360–2310 cm^−1^, attributed to asymmetric stretching vibrations, and at 668 cm^−1^, associated with the degenerate bending vibrations of carbon dioxide, were mainly visible [63,64]. A small signal at 2190 cm^−1^ suggested a small amount of carbon monoxide, and the band at 968 cm^−1^ was related to the out-of-plane C–H deformation vibrations of the vinyl groups. Bands at 1250 and 1180 cm^−1^ were related to the stretching vibrations of C–O groups located in ester type gases [65]. They suggested the initial decomposition of ester functionalities in unreacted L-M. More gaseous products were released in the second step, where the mass loss was 85%. In this stage, the same bands were visible, but their intensity was much higher. At this temperature, the cross-linked parts of the biocomposites decomposed, and there was a significant evolution of carbon dioxide, with bands at ca. 2300 and 668 cm^−1^. Absorption bands at 1770 and 1732 cm^−1^ were assigned to carbonyl compounds such as esters, acids, aldehydes, and anhydrides. The signals at 1605 and 1510 cm^−1^ were specific for aromatic derivatives such as phenol, benzene, and toluene, resulting from the degradation of BA.GDA and lignin moieties. Bands at 1460–1300 cm^−1^ were attributed to deformation vibrations, and bands at 3000–2850 cm^−1^ were attributed to the stretching vibrations of CH, CH_2_, and CH_3_ groups in the chemical structures of unsaturated and saturated aliphatic hydrocarbons or aromatic compounds. The signals located between 3700 and 3200 cm^−1^ were assigned to water vapors and alcohols, which can appear during the thermal degradation of esters, secondary hydroxyls, or ether groups [66]. 

In the first decomposition stage of EHA + 10% L-M (Figure 3b) and St + 10% L-M, the signals were similar to those given by BA + 10% L-M, suggesting the initial thermal decomposition of unreacted L-M. For St + 10% L-M (Figure 3c) the signal intensity was lower, apart from the absorption band at 682 cm^−1^, which was related to the decomposition of the unreacted styrene [16]. In the second decomposition stage of EHA + 10% L-M, the increase in signal intensity of –OH stretching vibrations may have been related to differences in chemical structure between EHA and BA. The second decomposition stage of St + 10% L-M contained signals in the 3100–3000 cm^−1^ range along with a stronger signal from styrene at 682 cm^−1^ [16] and a new signal at 769 cm^−1^ from the C–H deformation vibrations of aromatic rings. There were no bands in the 3000–2850 cm^−1^ region. These observations suggested the formation of aromatic compounds and their derivatives, rather than the formation of aliphatic compounds.

**Figure 4:**
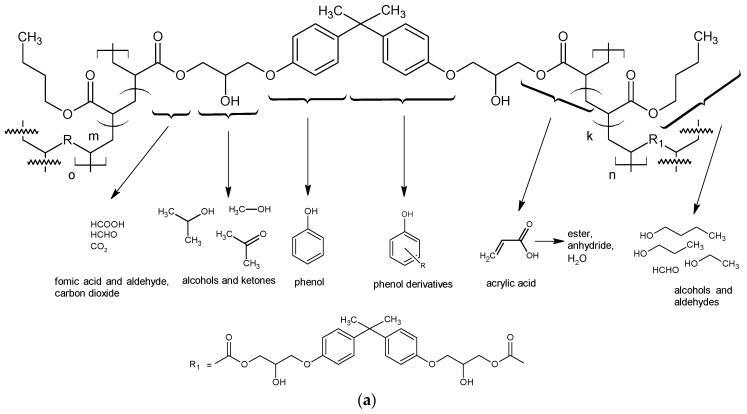

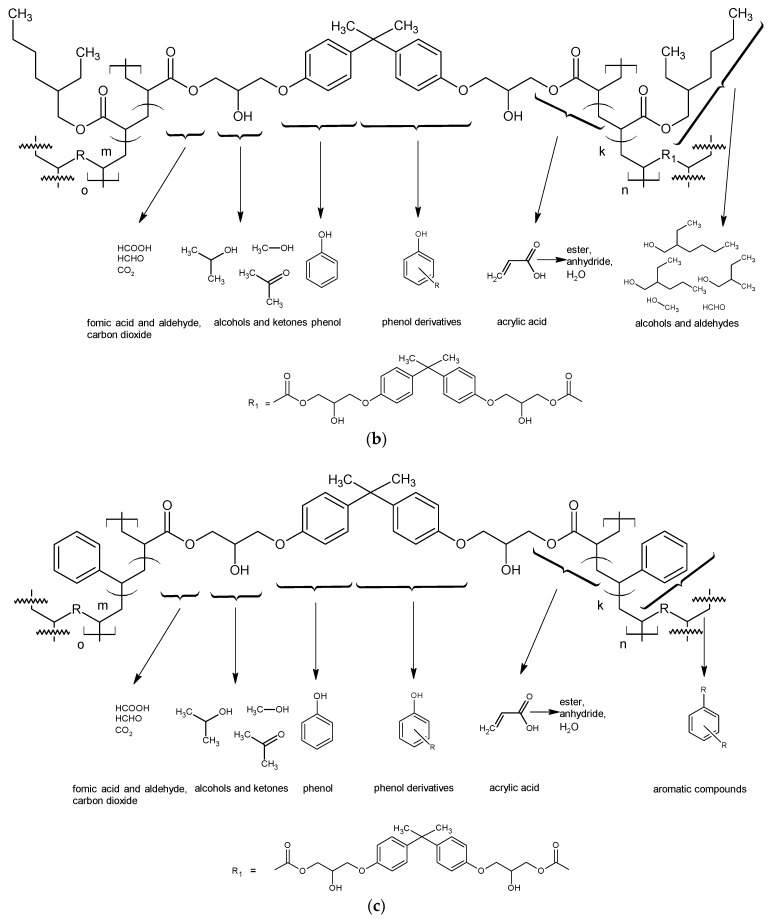
Proposed schemes of polymeric network fragments and possible thermal decomposition products of (**a**) biocomposites with BA, (**b**) biocomposites with EHA, and (**c**) biocomposites with St.

Schemes of the polymeric network fragments with the possible thermal decomposition products of the composites are proposed in Figure 4a–c. Fragments of lignin and possible thermal decomposition products are described in [61].

### 3.3. Mechanical and Thermomechanical Characterization

The Shore hardness values of the composites are presented in Table 3. The composites containing St had the highest Shore hardness values. The values with acrylates (BA and EHA) were lower and similar. In all cases, the addition of lignin increased the hardness, the highest values being obtained for the biocomposites with 5% lignin, where lignin could participate in the polymer network. When its role was solely as a filler, the hardness value was lower. The results were in a good agreement with those from previous analyses (DSC and TG/DTG). 

The results of mechanical tests on the materials without lignin and with 10% lignin (L/L-M) are presented in Table 4. As expected, the results of uniaxial stretching tests showed that in all cases, the addition of unmodified lignin caused a decrease in the elastic modulus (Young’s modulus). The smallest change was observed in the St material. Comparing St and St + 10% L, the ultimate stress decreased by approximately 50%. The largest change in Young's modulus from 1620 to 599 MPa and in the ultimate stress from 31.8 to 9.7 MPa was shown by the addition of 10% L to the EHA sample. Such a great decrease may mean that products made of this material showed too little resistance to external forces. The three-point bending test confirmed that lignin reduced the ultimate strength of the sample. The bending strength decreased by approximately 45% when lignin was added, and the EHA samples again showed the greatest decrease from 62.5 to 32.2 MPa. The data presented here showed that mechanical properties of the studied copolymers were changed accordingly to the active diluents used [67]. The strongest copolymers were those containing aromatic styrene, and the weakest were those with aliphatic, branched EHA as the active diluent.

Among the composites with L-M, those with styrene in the cross-section had a layered structure with two layers in the upper and lower regions with greater strength and stiffness, as well as a clear core with a coherent structure and defined flexibility. This was due to the fact that samples with modified lignin (L-M) did not fully polymerize over the entire cross-section. The copolymers of styrene and modified lignin polymerized only in thin layers, and the addition of a thermal initiator did not improve this process, as indicated by the results of tests under uniaxial tensile conditions, where the stress at break was 7.1–9.2 MPa. In bending, however, the strength was greater, in the range of 20.1–29.9 MPa.

**Figure 5:**
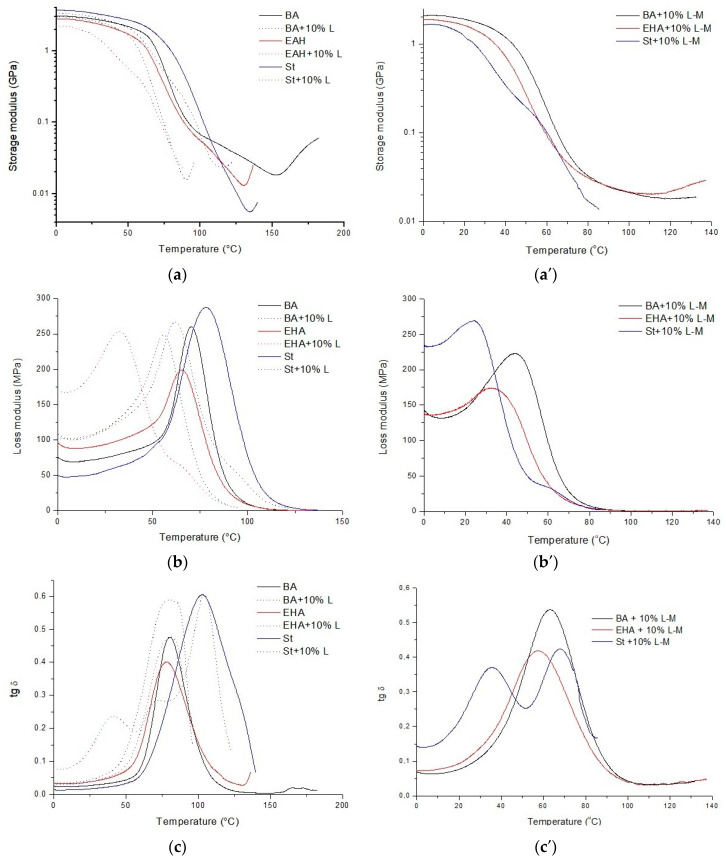
Storage modulus (**a**,**a′**), loss modulus (**b**,**b′**), and damping factor (tan delta) (**c**,**c′**) versus temperature for composites without lignin (BA, EHA, and St) and with 10% lignin (L-M/L).

In order to conduct a deeper analysis, additional DMA tests were carried out on materials without lignin and with 10% unmodified lignin with straining at different temperatures to reveal their viscoelastic properties. These results are shown in Figure 5 and Table 5. The storage modulus determined in the glassy region was higher in the materials without lignin then in the systems loaded with filler. Despite the use of a compound containing two unsaturated bonds (BPA.GDA) and reactive diluents enabling the formation of a polymer network (BA, EHA, and St)), no plateau in the rubbery region characteristic of a cross-linked materials was observed. In fact, after passing through a minimum value in this region, the storage modulus increased slightly with increasing temperature, particularly in systems containing filler. This suggested that the unmodified lignin hindered the polymerization process, an idea supported by the DSC results that showed exothermic effects associated with post-crosslinking. They were greater for composites with unmodified lignin. The influence of the filler on the degree of polymerization was also evident in the values of the loss modulus (E″). The glass transition temperature (*T*_g_) derived from the maximum loss modulus was highest with St and lowest with EHA. Among the composites with unmodified lignin, the EHA system showed the greatest drop in glass transition temperature at 32.3 °C. The effect depended on the type of active diluent used, which copolymerized in different ways with BPA.GDA. In the case of EHA + 10% L, the difference may have been due to the two-stage glassy transition observed in the two maxima in tan delta. A similar effect was observed in the system with St, but two tan delta maxima could not be distinguished due to the overlap of the glass transitions. The wide tan delta in the case of BA + 10% L may also have been associated with the overlap of two glass transitions, but in this case, the difference was smaller. 

Due to the difficulty in the polymerization of the compounds with modified lignin, the composites showed slightly lower parameters from the DMA data (Figure 5a′–c′). Only in the case of the EHA-based material did the addition of methacrylated lignin not cause a significant change in the storage modulus (E′) and in the glass transition temperatures (*T*_g_). Aromatic styrene had a very low tendency to UV polymerization, which occurred only in the outer layers reached by the UV radiation. The deeper layers were not cured, even in the presence of a thermal initiator. 

**Figure 6:**
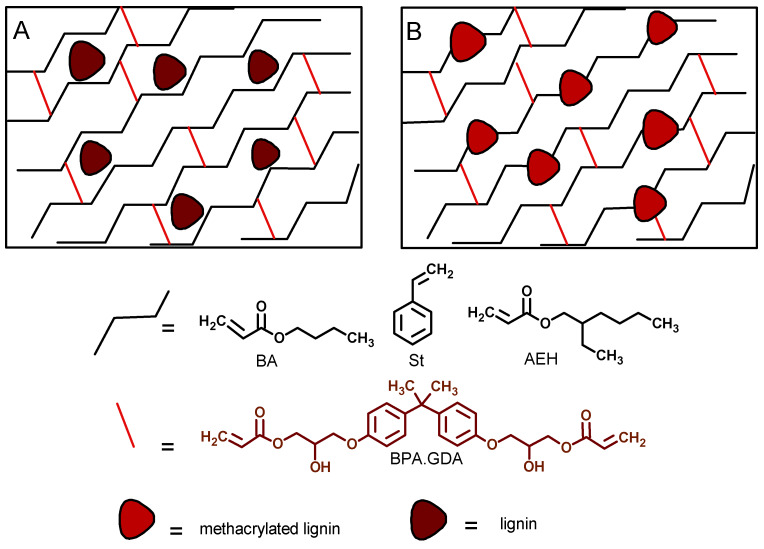
Proposed scheme of composite structure.

Based on the structural, thermal, and mechanical properties of the obtained composites, we believe that the unmodified lignin mainly acted as filler in the composite without any significant effect on the UV-polymerization of the polymeric matrix (Figure 6A). In contrast, the methacrylated lignin took part in the UV-curing process and was chemically integrated into the biocomposite structure, as illustrated in Figure 6B.

## 4. Conclusions

Lignin-containing polymer biocomposites were obtained by a UV-curing process with bisphenol A glycerolate (1 glycerol/phenol) diacrylate (BPA.GDA) as the main monomer and butyl acrylate (BA), 2-ethylhexyl acrylate (EHA) or styrene (St) as the reactive diluent. 

When 5 wt % L-M was added, it participated in the creation of a polymer network with improved thermal properties, but when the amount of L-M was increased to 10 wt %, the unreacted lignin derivative resulted in exothermic effects in the DSC curves, indicating a continuing polymerization process. In the biocomposites with unmodified lignin (L), the lignin mainly acted as a filler and interactions between the hydroxyl groups of the lignin and the carbonyl groups of acrylates seemed to occur. When St was added as a reactive diluent, the biocomposites had a more homogeneous structure than those to which acrylate monomers were added. Among the composites with acrylate monomers, which were less reactive than those with the styrene monomer, homopolymerization and cyclization processes could take place, and this resulted in a greater material heterogeneity. St-containing composites were characterized by higher thermal resistance that could have been related to the aromatic structure of this monomer. 

The addition of lignin increased the Shore D hardness value, especially when 5% of lignin was added to the composite material. The increase was more significant when L-M was used and for St-containing materials. The highest mechanical strength was observed in the copolymers of aromatic styrene, and the lowest mechanical strength was observed when the active diluent was aliphatic, branched EHA. The DMA analysis showed that the use of lignin as a filler influenced the polymerization process. Complete UV-curing was possible only in thin layers, which may indicate the potential application of UV-curing coatings. 

The incorporation of lignin and its methacrylic derivatives into polymer composites can promote the sustainability of the plastics industry and contribute to the better utilization of lignin.

## Figures and Tables

**Table 1:** Parameters of the syntheses. BPA.GDA: bisphenol A glycerolate (1 glycerol/phenol) diacrylate; BA: butyl acrylate; EHA: 2-ethylhexyl acrylate; St: styrene; L: unmodified lignin; L-M: methacrylated lignin.

Composite	BPA.GDA (g)	BA/EHA/St (g)	L/L-M (wt %)	L/L-M (g)
BA	4.652	1.396	0	0
BA + 5% L-M	4.421	1.326	5	0.287
BA + 5% L	4.365	1.310	5	0.284
BA + 10% L-M	4.287	1.286	10	0.557
BA + 10% L	4.438	1.331	10	0.577
EHA	4.588	1.376	0	0
EHA + 5% L-M	4.498	1.349	5	0.292
EHA + 5% L	4.423	1.327	5	0.287
EHA + 10% L-M	4.364	1.309	10	0.567
EHA + 10% L	4.219	1.266	10	0.548
St	4.579	1.374	0	0
St + 5% L-M	4.387	1.316	5	0.285
St + 5% L	4.441	1.332	5	0.289
St + 10% L-M	4.497	1.349	10	0.585
St + 10% L	4.499	1.350	10	0.585

**Table 2:** TG/DTG data for composites without lignin (BA, EHA and St) and with 10% lignin (L-M/L). *T*_5%_ and *T*_50%_: temperatures of 5% and 50% mass loss, respectively; *m*_loss1_: first mass loss; *m*_loss2_: second mass loss; RM: residual mass.

Composite	*T*_5%_ (°C)	*T*_50%_ (°C)	*T*_max1_ (°C)	*T*_max2_ (°C)	*m*_loss1_ (%)	*m*_loss2_ (%)	RM (%)
BA	349	418	-	418	-	92.81	7.19
BA + 10% L-M	182	413	176	418	8.01	83.14	8.85
BA + 10% L	290	409	-	410	-	85.21	14.79
EHA	362	425	-	425	-	93.65	6.35
EHA + 10% L-M	178	411	191	417	11.47	80.26	8.27
EHA + 10% L	185	406	186	411	8.64	78.08	13.28
St	344	418	-	422	-	96.20	3.80
St + 10% L-M	220	415	164	423	5.87	86.60	7.53
St + 10% L	269	414	-	419	-	90.18	9.82

**Table 3:** Shore hardness of the composites.

Composite	Shore Hardness Mean (°Sh)
BA	66.3 ± 2.1
BA + 5% L-M	80.3 ± 4.5
BA + 5% L	77.3 ± 3.8
BA + 10% L-M	74.0 ± 5.9
BA + 10% L	69.2 ± 5.1
EHA	68.7 ± 2.0
EHA + 5% L-M	81.0 ± 4.2
EHA + 5% L	75.5 ± 3.5
EHA + 10% L-M	77.2 ± 4.8
EHA + 10% L	70.0 ± 4.0
St	78.1 ± 1.0
St + 5% L-M	97.3 ± 2.2
St + 5% L	90.5 ± 3.1
St + 10% L-M	88.4 ± 3.8
St + 10% L	80.2 ± 3.3

**Table 4:** Results of mechanical tests on composites without lignin (BA, EHA and St) and with 10% lignin (L-M and L).

Composite	Stress at Break (MPa)	Relative Elongation at Break (%)	Young’s Modulus (MPa)
Tensile tests			
St	46.2	3.1	1890
St + 10% L	21.1	1.7	1590
St + 10% L-M	7.1	0.8	964
EHA	31.8	3.2	1620
EHA + 10% L	9.7	1.3	599
EHA + 10% L-M	8.3	1	1120
BA	49.9	2.8	2160
BA + 10% L	18.5	1.9	1300
BA + 10% L-M	9.2	1.2	1215
Bending tests			
St	78.2	1.7	4210
St + 10% L	51.3	2.1	3680
St + 10% L-M	20.1	1.5	1430
EHA	62.5	2.2	2590
EHA + 10% L	32.2	2.2	1520
EHA + 10% L-M	26.2	1.8	1488
BA	83.2	2.7	2901
BA + 10% L	53.9	1.9	2840
BA + 10% L-M	29.9	1.8	1544

**Table 5:** Dynamic mechanical analyzer (DMA) data for composites without lignin (BA, EHA, and St) and with 10% lignin (L-M/L).

Properties	Composites								
	BA	BA + 10% L	BA +10% L-M	EHA	EHA + 10% L	EHA + 10% L-M	St	St + 10% L	St + 10% L-M
E’(25 °C)/GPa	2.82	2.65	1.77	2.49	1.70	1.45	3.39	3.00	1.0
T_g_/°C ^a^	70.3	55.6	44.3	65.2	32.5	32.3	78.0	61.9	24.5/62.2

^a^ Determined from loss modulus max.

## Supplementary Materials

The following are available online at https://www.mdpi.com/2073-4360/12/5/1159/s1, Figure S1: ATR/FT-IR spectra of: (a) biocomposites with BA, (b) biocomposites with EHA, (c) biocomposites with St, Figure S2: DSC curves of: (a) BA/EHA/St and 10% L-M before polymerization (polymerization takes place during the analysis), (b) BA/EHA/St biocomposites with 10% L, (c) BA/EHA/St biocomposites with 10% L-M, Figure S3: (a) TG and (b) DTG curves of BA, BA + 10% L-M and BA + 10% L composites, Figure S4: (a) TG and (b) DTG curves of EHA, EHA + 10% L-M and EHA + 10% L composites, Figure S5: (a) TG and (b) DTG curves of St, St + 10% L-M and St + 10% L composites.

## References

[1] Laskoski M., Dominguez D.D., Keller T.M. (2005). Synthesis and properties of a bisphenol a based phthalonitrile resin. J. Polym. Sci. A.

[2] Derradji M., Ramdani N., Zhang T., Wang J., Gong L.D., Xu X.D., Lin Z.W., Henniche A., Rahoma H.K.S., Liu W.B. (2016). Effect of silane surface modified titania nanoparticles on the thermal, mechanical, and corrosion protective properties of a bisphenol-A based phthalonitrile resin. Prog. Org. Coat..

[3] Zhang D., Jia D. (2006). Toughness and strength improvement of diglycidyl ether of bisphenol-A by low viscosity liquid hyperbranched epoxy resin. J. Appl. Polym. Sci..

[4] Iyer S., Schiraldi D.A. (2007). Role of Specific Interactions and Solubility in the Reinforcement of Bisphenol A Polymers with Polyhedral Oligomeric Silsesquioxanes. Macromolecules.

[5] Skrtic D., Antonucci J.M., Liu D.W. (2006). Ethoxylated bisphenol dimethacrylate-based amorphous calcium phosphate composites. Acta Biomater..

[6] Imai Y., Terahara A., Hakuta Y., Matsui K., Hayashi H., Ueno N. (2009). Transparent poly(bisphenol A carbonate)-based nanocomposites with high refractive index nanoparticles. Eur. Polym. J..

[7] Cao L., Liu X., Na H., Wu Y., Zheng W., Zhu J. (2013). How a bio-based epoxy monomer enhanced the properties of diglycidyl ether of bisphenol A (DGEBA)/graphene composites. J. Mater. Chem. A.

[8] Jang L.W., Lee D.C. (2000). Polystyrene/bisphenol A polycarbonate molecular composite by in situ polymerization. I. Preparation and characterization. Polymer.

[9] Zhang X., He Q., Gu H., Colorado A., Wei S., Guo Z. (2013). Flame-retardant electrical conductive nanopolymers based on bisphenol F epoxy resin reinforced with nano polyanilines. ACS Appl. Mater. Interfaces.

[10] Lin C.T., Lee H.T., Chen J.K. (2015). Preparation and properties of bisphenol-F based boron-phenolic resin/modified silicon nitride composites and their usage as binders for grinding wheels. Appl. Surf. Sci..

[11] Chen X., Jiao C., Li S., Sun J. (2011). Flame retardant epoxy resins from bisphenol-A epoxy cured with hyperbranched polyphosphate ester. J. Polym. Res..

[12] Guzel G., Deveci H. (2018). Properties of polymer composites based on bisphenol A epoxy resins with original/modified steel slag. Polym. Compos..

[13] Wang J., Li Y., Huang M., Fang J., Wang C. (2014). Silicon–aluminum synergistic mechanism in flame *retardancy* of epoxy resin. Polym. Compos..

[14] Zhang J., Qi S. (2014). Mechanical, thermal, and dielectric properties of aluminum nitride/glass fiber/epoxy resin composites. Polym. Compos..

[15] Kang J.H., Kondo F., Katayama Y. (2006). Human exposure to bisphenol A. Toxicology.

[16] Cardona F., Rogers D., Davey S., Van Erp G. (2007). Investigation of the Effect of Styrene Content on the Ultimate Curing of Vinylester Resins by TGA-FTIR. J. Compos. Mater..

[17] Jaikumar V., Kumar D. (2015). New UV-Curable Prepolymer: Synthesis, Characterization, and Kinetics Analysis. Int. J. Polym. Anal. Charact..

[18] Koelewijn S.F., Van Den Bosch S., Renders T., Schutyser W., Lagrain B., Smet M., Thomas J., Dehaen W., Van Puyvelde P., Witters H. (2017). Sustainable bisphenols from renewable softwood lignin feedstock for polycarbonates and cyanate ester resins. Green Chem..

[19] Klapiszewski Ł., Madrawska M., Jesionowski T. (2012). Preparation and characterisation of hydrated silica/lignin biocomposites. Physicochem. Probl. Miner. Process..

[20] Salasinska K., Barczewski M., Górny R., Kloziński A. (2018). Evaluation of highly filled epoxy composites modified with walnut shell waste filler. Polym. Bull..

[21] Barczewski M., Sałasińska K., Szulc J. (2019). Application of sunflower husk, hazelnut shell and walnut shell as waste agricultural fillers for epoxy-based composites: A study into mechanical behavior related to structural and rheological properties. Polym. Test..

[22] Ojha S., Raghavendra G., Acharya S.K. (2014). A comparative investigation of bio waste filler (wood apple-coconut) reinforced polymer composites. Polym. Compos..

[23] Heriyanto F., Pahlevani V., Sahajwalla V. (2019). Effect of different waste filler and silane coupling agent on the mechanical properties of powder-resin composite. J. Clean. Prod..

[24] Harahap H., Sukardi A., Rusli I., Taslim I., Surya I. (2016). Effect of Microcrystalline Cellulose from Cassava Peel Waste Filler Loading on Natural Rubber Latex Products. J. Polym. Mater..

[25] Salasinska K., Polka M., Gloc M., Ryszkowska J. (2016). Composites with pistachio shell and sunflower husk: The effect of filler content and chemical constitution on the dimensional and fire stability. Polimery.

[26] Thakur V.K., Thakur M.K., Raghavan P., Kessler M.R. (2014). Progress in green polymer composites from lignin for multifunctional applications: A review. ACS Sustain. Chem. Eng..

[27] Berlin A., Balakshin M., Vijai G., Maria Tuohy G., Kubicek C.P., Saddler J., Xu F. (2014). Industrial lignins: Analysis, properties, and applications. Bioenergy Research: Advances and Application.

[28] Settle M., Lange H., Crestini C. (2013). Quantitative HSQC analyses of lignin: A practical comparison. Comput. Struct. Biotechnol. J..

[29] Mattsson C., Andersson S.I., Belkheiri T., Åmand L.E., Olausson L., Vamling L., Theliander H. (2016). Using 2D NMR to characterize the structure of the low and high molecular weight fractions of bio-oil obtained from LignoBoost™ kraft lignin depolymerized in subcritical water. Biomass Bioenergy.

[30] Crestini C., Lange H., Sette M., Argyropoulos D.S. (2017). On the structure of softwood kraft lignin. Green Chem..

[31] Crestini C., Argyropoulos D.S. (1997). Structural Analysis of Wheat Straw Lignin by Quantitative ^31^P and 2D NMR Spectroscopy. The Occurrence of Ester Bonds and α-O-4 Substructures. J. Agric. Food Chem..

[32] Hatakeyama H., Hatakeyama T., Abe A., Dusek K., Kobayashi S. (2009). Lignin Structure, Properties, and Applications. Biopolymers. Advances in Polymer Science.

[33] Adler E. (1957). Structural Elements of Lignin. Ind. Eng. Chem..

[34] Onnerud H., Gellerstedt G. (2003). Inhomogeneities in the chemical 696 structure of spruce lignin. Holzforschung.

[35] Rencoret J., Kim H., Evaristo A.B., Gutiérrez A., Ralph J., Del Río J.C. (2018). Variability in Lignin Composition and Structure in Cell Walls of Different Parts of Macaúba (*Acrocomia aculeata*) Palm Fruit. J. Agric. Food Chem..

[36] Vishtal A., Kraslawski A. (2011). Challenges in industrial applications of technical lignins. Bioresources.

[37] Svärd A., Sevastyanova O., Dobele G., Jurkjane V., Brännvall E. (2016). COST Action FP1105: Effect of raw materials and pulping conditions on the characteristics of dissolved kraft lignins. Holzforschung.

[38] Balakshin M.Y., Capanema E.A., Chang H.M., Hu T.Q. (2008). Recent advances in the isolation and analysis of lignins and lignin-carbohydrate complexes. Characterization of Lignocellulosic Materials.

[39] Hatakeyama H., Hatakeyama T. (2010). Lignin structure, properties, and applications. Adv. Polym. Sci..

[40] Lora J.H., Glasser W.G. (2002). Recent industrial applications of lignin: A sustainable alternative to nonrenewable materials. J. Polym. Environ..

[41] Košíková B., Gregorová A., Osvald A., Krajčovičová J. (2007). Role of lignin filler in stabilization of natural rubber–based composites. J. Appl. Polym. Sci..

[42] Strzemiecka B., Klapiszewski Ł., Jamrozik A., Szalaty T., Matykiewicz D., Sterzynski T., Voelkel A., Jesionowski T. (2016). Physicochemical Characterization of Functional Lignin-Silica Hybrid Fillers for Potential Application in Abrasive Tools. Materials.

[43] Sun J., Wang C., Yeo J.C.C., Yuan D., Li H., Stubbs L.P., He C. (2016). Lignin Epoxy Composites: Preparation, Morphology, and Mechanical Properties. Macromol. Mater. Eng..

[44] Goliszek M., Podkościelna B., Sevastyanova O., Fila K., Chabros A., Pączkowski P. (2019). Investigation of accelerated aging of lignin-containing polymer materials. Int. J. Biol. Macromol..

[45] Wood B.M., Coles S.R., Maggs S., Meredith J., Kirwan K. (2011). Use of lignin as a compatibiliser in hemp/epoxy composites. Compos. Sci. Technol..

[46] Sen S., Patil S., Argyropoulos D.S. (2015). Thermal Properties of Lignin in Copolymers, Blends, and Composites; A Review. Green Chem..

[47] Hajirahimkhan S., Xu C.C., Ragogna P.J. (2018). Ultraviolet Curable Coatings of Modified Lignin. ACS Sustain. Chem. Eng..

[48] Podkościelna B., Goliszek M., Sevastyanova O. (2017). New approach in the application of lignin for the synthesis of hybrid materials. Pure Appl. Chem..

[49] Goliszek M., Podkościelna B. (2019). Synthesis and characterization of polymer biocomposites with lignin. Physicochem. Probl. Miner..

[50] Srinivasa R.Y., Kollipara P. (2016). Preparation and characterisation of lignin nanoparticles: Evaluation of their potential as antioxidants and UV protectants, *J*. Exp. Nanosci..

[51] Svobodova A., Psotova J., Walterova D. (2003). Natural phenolics in the prevention of UV-induced skin damage. A review. Biomed. Pap..

[52] Garcıa A., Toledano A., Andres M.A., Labidi J. (2010). Study of the antioxidant capacity of Miscanthus sinensis lignins, *Proc*. Biochem..

[53] Aminzadeh S., Lauberts M., Dobele G., Ponomarenko J., Mattsson T., Lindström M.E., Sevastyanova O. (2018). Membrane filtration of kraft lignin: Structural charactristics and antioxidant activity of the low-molecular-weight fraction. Ind. Crop. Prod..

[54] Dizhbite T., Telysheva G., Jurkjane V., Viesturs U. (2004). Characterization of the radical scavenging activity of lignins—Natural antioxidants. Bioresour. Technol..

[55] Lauberts M., Sevastyanova O., Ponomarenko J., Dizhbite T., Dobele G., Volperts A., Lauberte L., Telysheva G. (2017). Fractionation of technical lignin with ionic liquids as a method for improving purity and antioxidant activity. Ind. Crop. Prod..

[56] Tagami A., Gioia C., Lauberts M., Budnyak T., Moriana R., Lindström M.E., Sevastyanova O. (2019). Solvent fractionation of softwood and hardwood kraft lignins for more efficient uses: Compositional, structural, thermal, antioxidant and adsorption properties. Ind. Crop. Prod..

[57] Hamedani G., Ebrahimi M., Ghaffarian S. (2006). Synthesis and Kinetics Study of Vinyl Ester Resin in the Presence of Triethylamine. Iran. Polym. J..

[58] Lee Y.R., Kim S.C., Lee H., Jeong H.M., Raghu A.V., Reddy K.R., Kim B.K. (2011). Graphite oxides as effective fire retardants of epoxy resin. Macromol. Res..

[59] Kawasaki A., Furukawa J., Tsuruta T., Wasai G., Makimoto T. (1961). Infrared spectra of poly(butyl acrylates). Macromol. Chem. Phys..

[60] Gordobil O., Moriana R., Zhang L., Labidi J., Sevastyanova O. (2016). Assessment of technical lignins for uses in biofuels and biomaterials: Structure-related properties, proximate analysis and chemical modification. Ind. Crop. Prod..

[61] Sobiesiak M., Podkościelna B., Sevastyanova O. (2017). Thermal degradation behavior of lignin-modified porous styrene-divinylbenzene and styrene-bisphenol A glycerolate diacrylate copolymer microspheres. J. Anal. Appl. Pyrol..

[62] Brill R.P., Palmese G.R. (2000). An investigation of vinyl–ester-styrene bulk copolymerization cure kinetics using Fourier transform infrared spectroscopy. J. Appl. Polym. Sci..

[63] Spiridon I., Tanase C.E. (2018). Design, characterization and preliminary biological evaluation of new lignin-PLA biocomposites. Int. J. Biol. Macromol..

[64] Maciejewska M., Rogulska M. (2019). Insight into functionalized DMN-*co*-GMA copolymers. J. Therm. Anal. Calorim..

[65] Tudorachi N., Mustata F. (2017). Curing and thermal degradation of diglycidyl ether of bisphenol A epoxy resin crosslinked with natural hydroxy acids as environmentally friendly hardeners. Arab. J. Chem..

[66] Silverstein R.M., Webster F.X., Kiemle D.J. (2005). Spectrometric Identification of Organic Compounds.

[67] Podkościelna B. (2013). The influence of oxidation number of sulfur on the polymerization and themo- mechanical properties of dimethacrylate copolymers. J. Therm. Anal. Calorim..

