# Supplementary material for: Preparation, Thermal, and Mechanical Characterization of UV-Cured Polymer Biocomposites with Lignin
Source: Polymers (Basel). 2020 May 19;12(5):1159. doi: 10.3390/polym12051159 (PMC7285094; doi:10.3390/polym12051159)
Supplement: Supplementary file 1 [file polymers-12-01159-s001.pdf]

Supplementary Material

Preparation, Thermal and Mechanical Characterization of UV-Cured Polymer Biocomposites with Lignin

Marta Goliszek [1,2,*], Beata Podkościelna [1], Tomasz Klepka [3], Olena Sevastyanova [4,5]

[1] Department of Polymer Chemistry, Institute of Chemical Science, Faculty of Chemistry, Maria Curie-Sklodowska University, M. Curie-Sklodowska Sq. 5, 20-031 Lublin, Poland; beatapod@poczta.umcs.lublin.pl
[2] Analytical Laboratory, Institute of Chemical Science, Faculty of Chemistry, Maria Curie-Sklodowska University, M. Curie-Sklodowska Sq. 5, 20-031 Lublin, Poland
[3] Department of Technology and Polymer Processing, Faculty of Mechanical Engineering, Lublin University of Technology, Nadbystrzycka 36, 20-618 Lublin, Poland; t.klepka@pollub.pl
[4] Department of Fibre and Polymer Technology, KTH Royal Institute of Technology, Teknikringen 56-58, SE-10044, Stockholm, Sweden; olena@kth.se
[5] Wallenberg Wood Science Center (WWSC), Department of Fibre and Polymer Technology, KTH Royal Institute of Technology, Teknikringen 56-58, SE-10044, Stockholm, Sweden

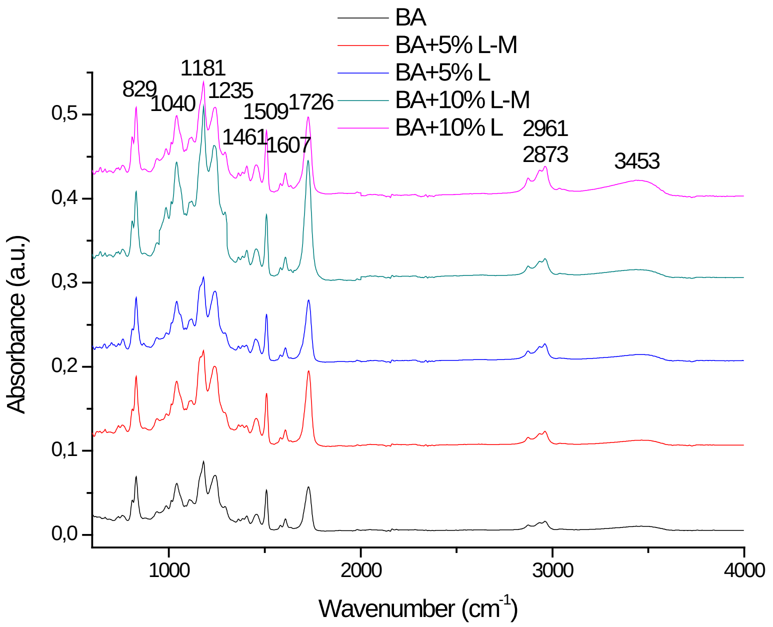

(a)

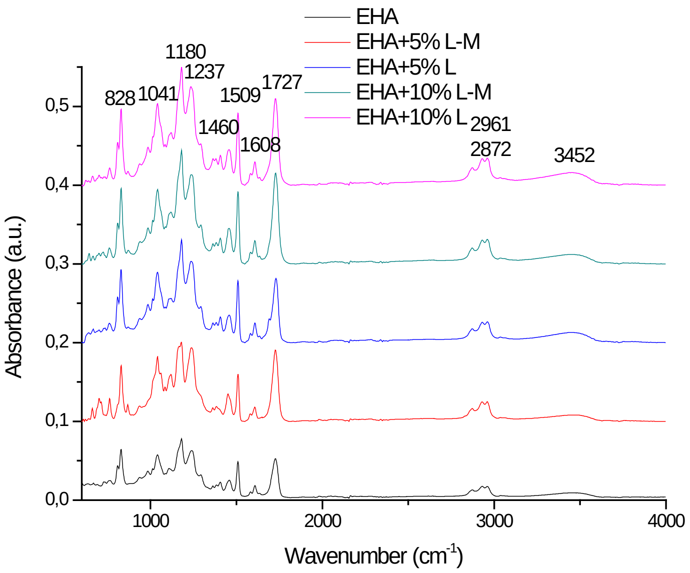

(**b**)

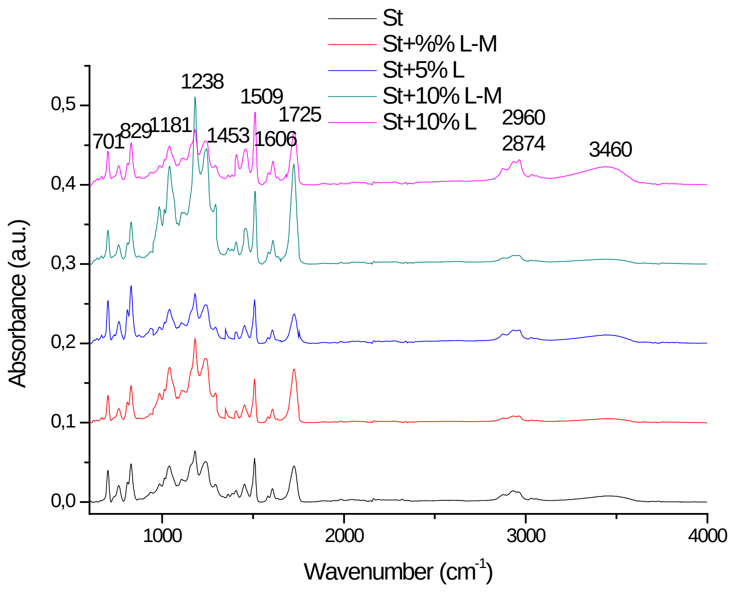

(**c**)

Figure S1. ATR/FT-IR spectra of: (**a**) biocomposites with BA, (**b**) biocomposites with EHA, (**c**) biocomposites with St.

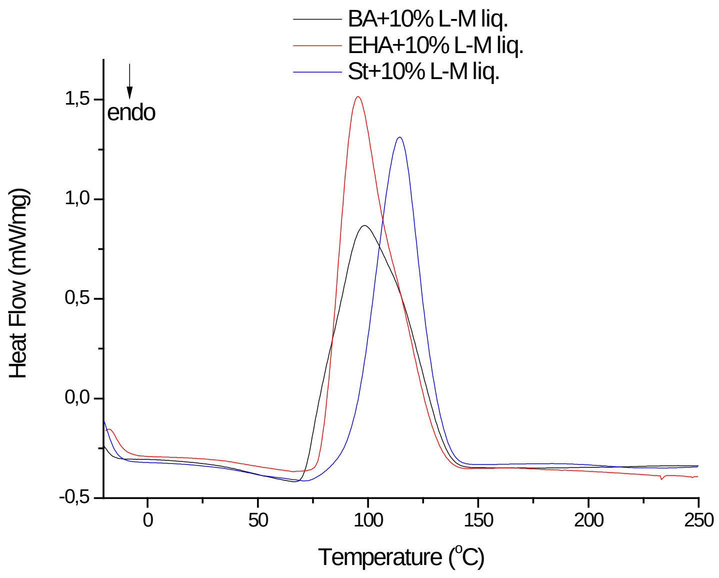
BA+10% L-M liq.
EHA+10% L-M liq.
St+10% L-M liq.
endo
Heat Flow (mW/mg)
Temperature (°C)
1,5
1,0
0,5
0,0
-0,5
0
50
100
150
200
250

(**a**)

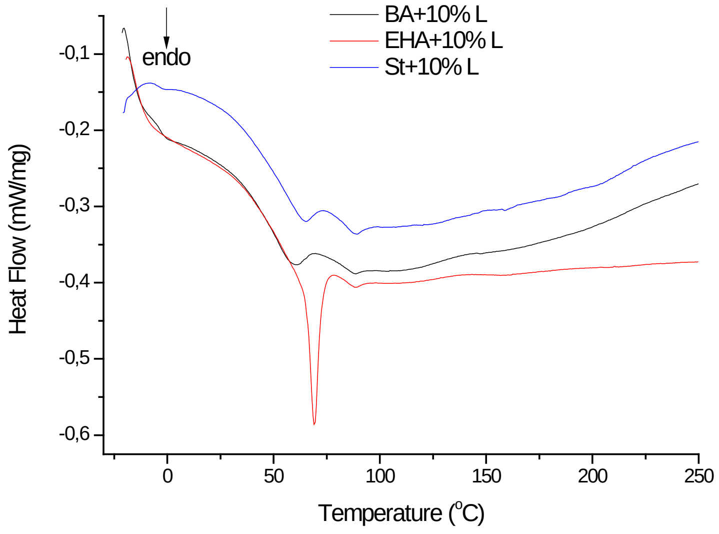
BA+10% L
EHA+10% L
St+10% L
endo
Heat Flow (mW/mg)
Temperature (°C)
-0,1
-0,2
-0,3
-0,4
-0,5
-0,6
0
50
100
150
200
250

(**b**)

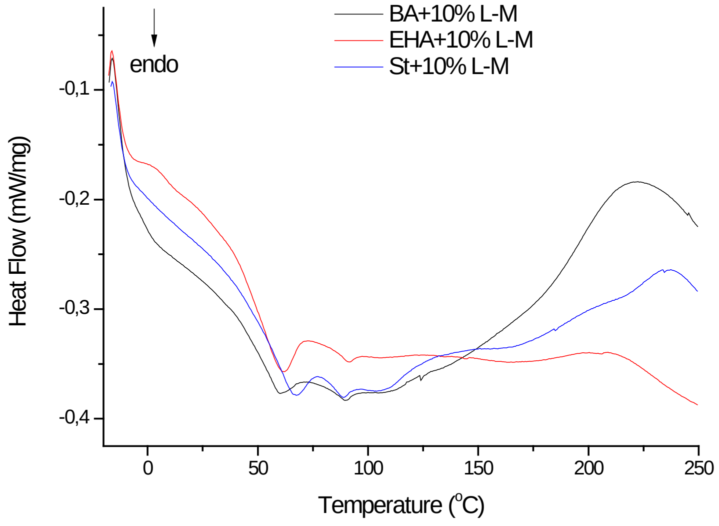

(**c**)

Figure S2. DSC curves of: (**a**) BA/EHA/St and 10% L-M before polymerization (polymerization takes place during the analysis), (**b**) BA/EHA/St biocomposites with 10% L, (**c**) BA/EHA/St biocomposites with 10% L-M.

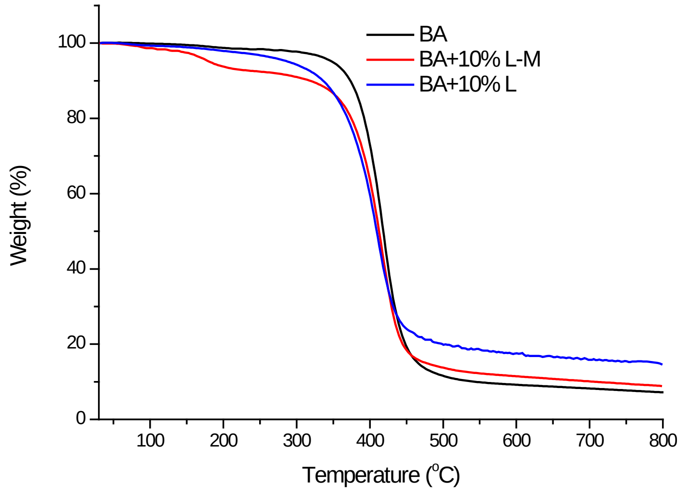

(**a**)

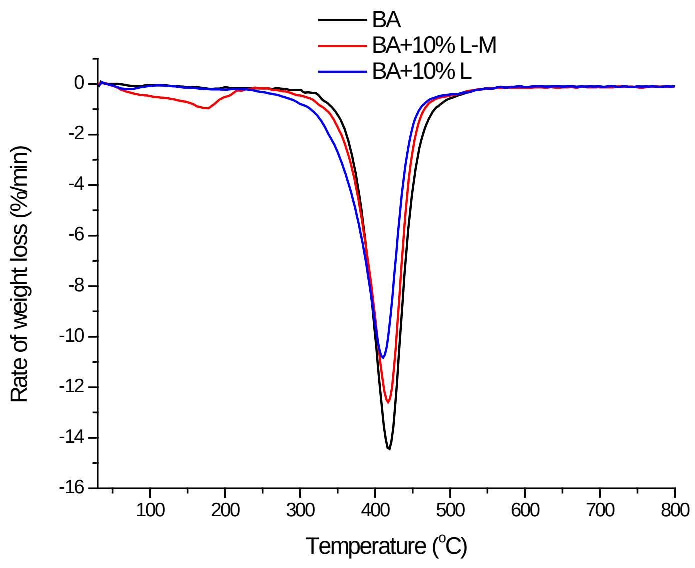

(**b**)

Figure S3. (**a**) TG and (**b**) DTG curves of BA, BA + 10% L-M and BA + 10% L composites.

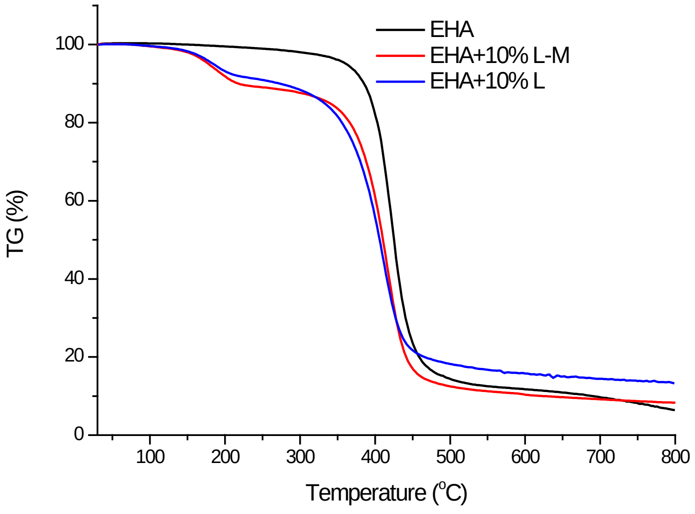

(**a**)

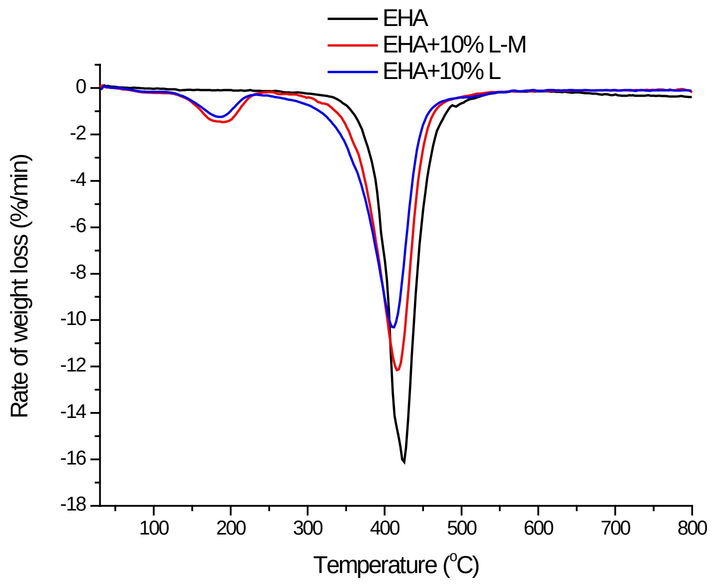

(**b**)

Figure S4. (**a**) TG and (**b**) DTG curves of EHA, EHA + 10% L-M and EHA + 10% L composites.

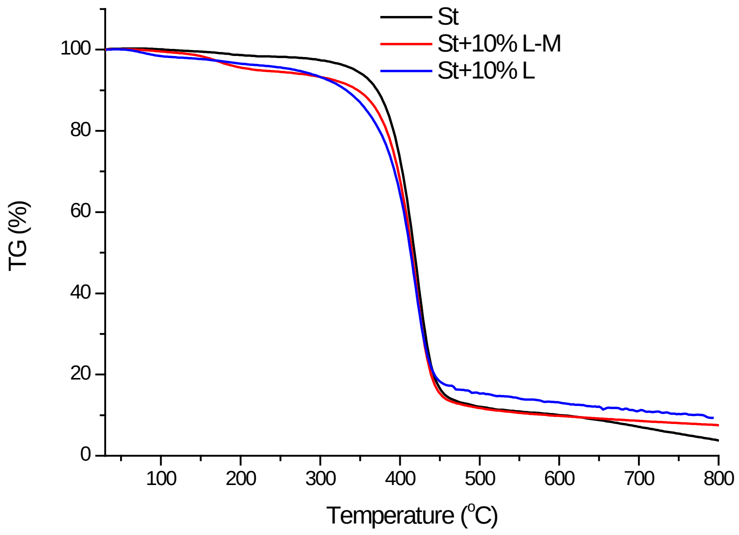

(**a**)

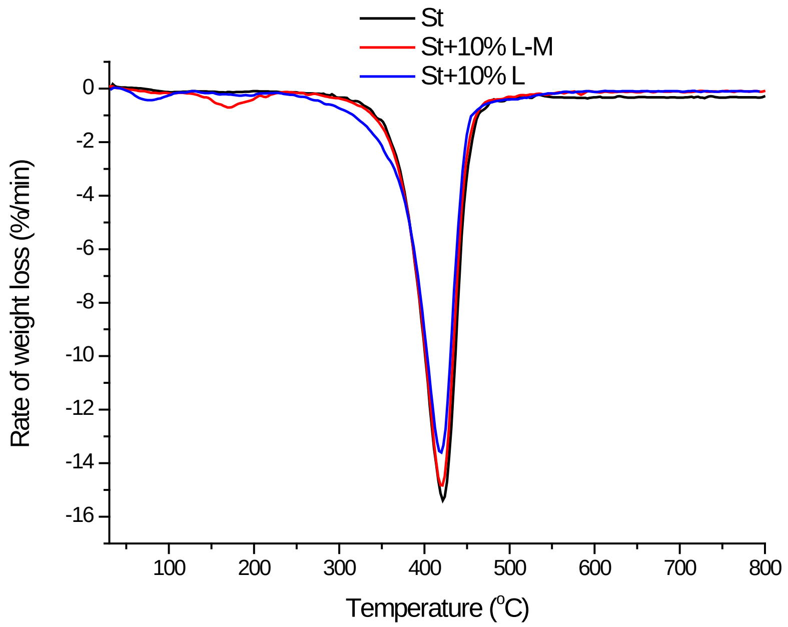

(**b**)

Figure S5. (**a**) TG and (**b**) DTG curves of St, St + 10% L-M and St + 10% L composites.
